# Signalling pathways and cellular functions of KDEL receptors: implications in cancer biology

**DOI:** 10.1007/s00018-025-05820-8

**Published:** 2025-08-06

**Authors:** Beatrice Dufrusine, Ilaria Cela, Chiara Gramegna Tota, Marta Palumbo, Michele Sallese

**Affiliations:** 1 Department of Bioscience and Technology for Food Agriculture and Environment, University of Teramo, Teramo, 64100 Italy; 2 Department of Innovative Technologies in Medicine and Dentistry, “G. d’ Annunzio” University of Chieti-Pescara, 66100 Chieti, Italy; 3 Center for Advanced Studies and Technology (CAST), “G. d’ Annunzio” University of Chieti-Pescara, Chieti, 66100 Italy

## Abstract

KDEL receptors (KDELRs) are a small family of seven-transmembrane domain proteins primarily localized to the membranes of the Golgi apparatus and endoplasmic reticulum (ER). These receptors are responsible for retrieving ER-resident chaperones that have trafficked to post-ER compartments. Beyond their primary role in retrieval, chaperone binding to KDELRs trigger diverse signalling pathways. These include the activation of protein kinase A, Src tyrosine kinase, and Rab1a/Rab3a that are mediated respectively by the α-subunits Gαs, Gαq, and Gαo of heterotrimeric G-proteins. KDELR-activated signalling pathways regulate intracellular transport of proteins and membranes, extracellular matrix (ECM) degradation, and the formation of membrane protrusions from the plasma membranes. More recently, crosstalk with the EGF receptor has been reported, offering a potential explanation for how chaperones, often overrepresented on the plasma membrane of cancer cells, may contribute to enhanced cell proliferation. Reflecting their established cellular roles, numerous studies have documented significant involvement of these receptors in a broad spectrum of cancers including colorectal cancer, breast tumours, glioblastoma, melanoma, chondrosarcoma, and lung adenocarcinoma. The strong association between KDELRs and cancer is further highlighted by the observed correlation between KDELR expression and immune cell infiltration in tumours. This effect may arise from the influence of KDELRs on the secretory pathway, alongside the immunomodulatory role of KDELR1 within immune cells. In conclusion, endomembrane-initiated signalling through KDELR plays a pivotal role in regulating fundamental cellular processes, maintaining physiological functions, and modulating key aspects of cancer biology.

## Introduction

KDEL receptors (KDELRs) are seven-transmembrane domain proteins of the PQ-loop protein family, predominantly located within the intracellular membranes of the endoplasmic reticulum (ER) and the Golgi complex [1–4]. More recently these receptors have also been reported in plasma membrane [5, 6]. The KDELRs were first identified through a genetic screening in *Saccharomyces cerevisiae*, aimed at finding proteins that could complement mutants defective in retaining proteins with the tetrapeptide HDEL at their C-terminus [7]. In fact, the yeast receptor is known as the HDEL receptor, or ERD2, which stands for ER retention defective 2. ER lumen of the yeast contains several enzymes and chaperones mostly terminating with an HDEL motif. These proteins can exit outside the ER for physiological or pathological reasons and move forward along the secretory pathway up to the Golgi complex where they bind to ERD2 receptor that retro-translocate them into the ER through coat protein complex I (COPI) vesicles. The same mechanism is conserved in vertebrates, although their genomes, including human, contain three genes for KDELRs, namely KDELR1, KDELR2 and KDELR3 [3]. Analogously to yeast, KDELRs derive their names from the most diffused ligand, which is the KDEL tetrapeptide present at the C-terminus of vertebrate ER chaperones, although these receptors bind a number of KDEL variants (e.g. HDEL, RDEL) [8]. More recently, the recycling paradigm has been challenged, suggesting that KDELR may function as a Golgi gatekeeper for chaperones rather than a transporter [9].

KDELRs are highly homologous, but each isoform preferentially binds a specific set of ligands [8, 10]. In vitro ligand-binding studies revealed that KDELRs specifically bind to KDEL-like sequences with a lower affinity compared to the canonical KDEL motif (KDEL > HDEL > DDEL > HDEV) [8]. A more recent study examining the interaction between KDEL variants and KDELR isoforms revealed a comprehensive map highlighting isoform-specific binding preferences for distinct KDEL sequence motifs [10]. Additionally, the selective relationship between chaperones and KDELRs is further emphasized by the upregulation of KDELR2 and KDELR3, but not KDELR1, in response to ER chaperone depletion caused by reduced ER calcium levels [10].

Interestingly, the affinity of KDELRs for KDEL ligands is influenced by pH. Indeed, the retrograde transport of proteins from the Golgi to the ER is driven by pH gradients between the two compartments. The acidic pH in the Golgi environment facilitates the binding of KDEL-bearing chaperones, while the neutral pH in the ER lumen promotes ligand dissociation from KDELRs [11, 12].

Our current understanding of the molecular pathways involving KDELRs, as well as their overall functions, remains incomplete. Part of the available knowledge refers to a generic KDELR, due to the absence of information on the specific isoform involved. In this review, we aim to first discuss the molecular pathways and cellular machineries linked to KDELR(s), and second, examine their associated cellular functions and involvement in cancer biology.

## KDEL receptor signalling and the ER stress response

To date, beyond their original chaperone-retrieval function, additional roles have been ascribed to KDELRs. These include their involvement in the unfolded protein response (UPR) and ER quality control, a mechanism ensuring that only properly folded proteins are exported from the ER [13, 14]. The UPR is stimulated by the accumulation of misfolded proteins in the ER and results in either cytoprotective or apoptotic effects [15].

In mammalian cells, the UPR is coordinated by three stress sensors: inositol-requiring protein 1α (IRE1), protein kinase RNA-like ER kinase (PERK), and activating transcription factor 6 (ATF6) [16–20]. These sensors are kept in an inactive state when bound to the ER chaperone binding immunoglobulin protein (BiP) [21]. An increase in misfolded proteins causes BiP to dissociate from these sensors, due to its higher affinity for misfolded or unfolded proteins, and activation of the UPR [17]. UPR aim at restoring the ER homeostasis through signalling and transcriptional reprogramming, which lead to reduction of protein synthesis, upregulation of chaperones and removal of misfolded proteins via the ER-associated protein degradation (ERAD) and autophagy. When these actions cannot resolve ER stress, UPR triggers cell death [15].

In 2003, Yamamoto et al. discovered that KDELR activates p38 mitogen-activated protein kinases in response to ER chaperone upregulation and leakage following tunicamycin treatment [13]. A mutant KDELR (R169N), unable to bind chaperones, failed to activate the kinase, thereby exacerbating the stress response and promoting cell death. This suggests that both signalling and retrieval activity are essential KDELR functions for managing ER stress. The involvement of KDELRs in the UPR is further supported by the upregulation of KDELR2 and KDELR3, but not KDELR1, in cells treated with UPR inducers such as tunicamycin and thapsigargin [10]. This effect seems to be mediated by the IRE1/XBP1 pathway [10].

## G protein-based KDEL receptor signalling

**Fig. 1:**
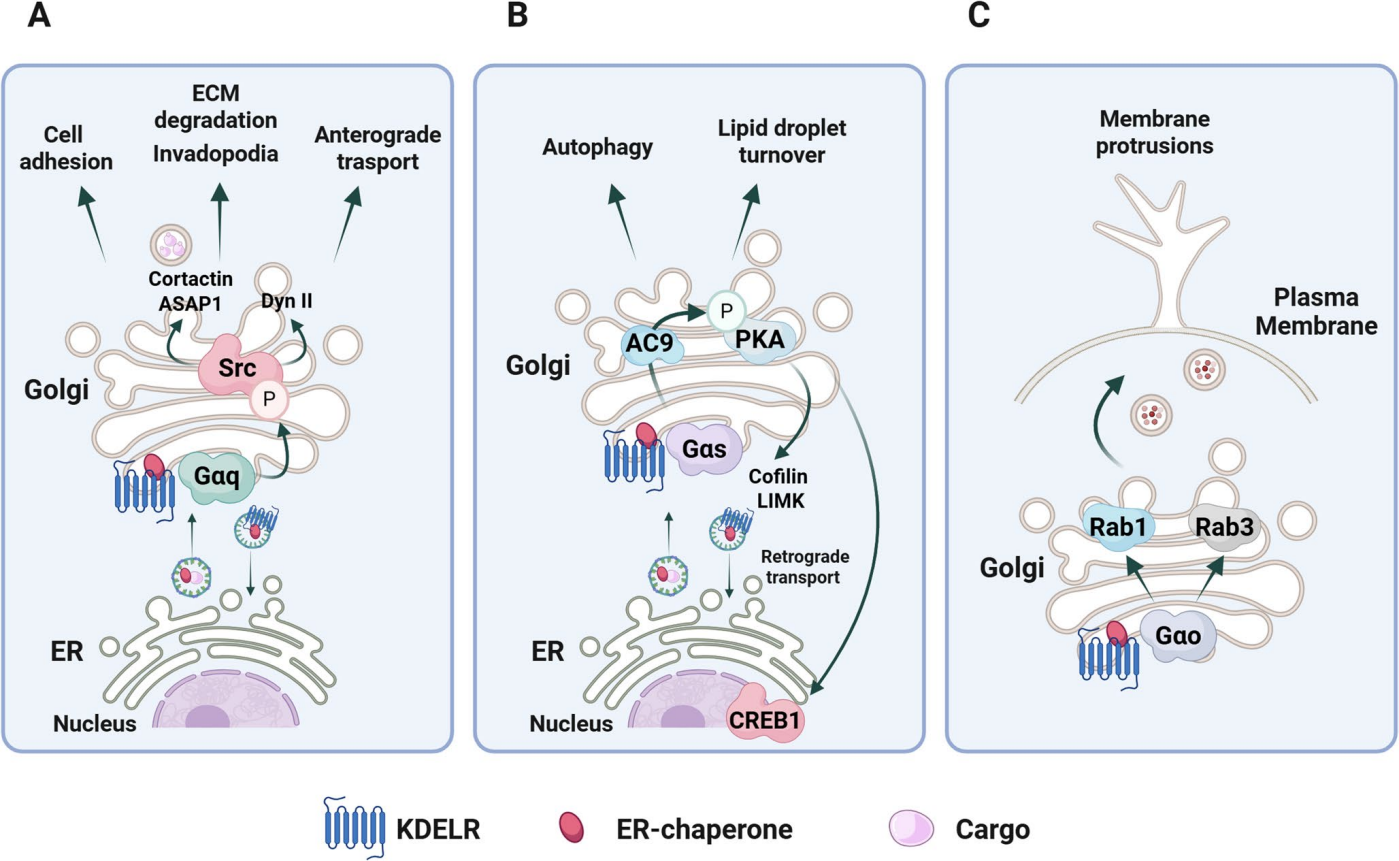
Schematic representation of KDELR signalling pathways and associated cellular processes. KDELR interacts with different G proteins (Gαq, Gαs, and Gαo) to activate distinct downstream effectors. Gαq-mediated Src activation regulates anterograde transport, cell adhesion, invadopodia formation, and ECM degradation. Gαs activates PKA, impacting retrograde transport, actin remodelling, autophagy, and lipid droplet turnover. Gαo leads to Rab1a/Rab3a activation, promoting membrane protrusion

In 2008, based on the idea that ER-resident chaperones might physiologically reach post-ER compartments and then return via KDELRs, we synchronized cargo transport along the secretory pathway to deliver a bolus of ER-resident chaperones to the KDELRs on the Golgi complex. The application of this traffic pulse led to a partial relocalization of KDELRs from the Golgi apparatus to the ER and an increase in the phosphorylation of tyrosine residues on several proteins [22]. We demonstrated that KDELR is responsible for the activation of these phosphorylation events and identified Src kinase as one of the key proteins activated downstream of KDELRs (Fig. 1) [22]. Unfortunately, the identities of the other phosphoproteins involved in the phosphorylation cascade, as well as the specific KDELRs engaged, have not yet been identified. We hypothesized that this endomembrane signalling plays a role in coordinating the amount of cargo arriving at the Golgi with its subsequent export from the organelle (Fig. 1). Although, a few years before it was reported that Golgi-Src perturbs Golgi organization and promotes the redistribution of KDELR towards the ER [23]. From a molecular standpoint we showed that the Gαq/11 subunit of the heterotrimeric G proteins take part in KDELR-dependent Src activation (Fig. 1) [24]. Moreover, Golgi-Src can phosphorylate the GTPase Dynamin II, a key protein in the formation of carriers at the trans-Golgi network [25]. Notably, constitutive Src activation, frequently observed in cancer cells, leads to Golgi fragmentation, a phenomenon that can be reversed through treatment with Src inhibitors [25]. Src-Dyn II could be considered a sort of check point that activates the Golgi fissioning machinery to handle the right amount of arriving cargo [25]. However, we cannot exclude the possibility that it also modulates the activities of Golgi enzymes involved in post-translational modification of the transiting cargoes.

Later on we realized that KDELR-activated Src does not remain confined to the Golgi membranes but spreads to other cell regions including the focal adhesions and invadopodia (Fig. 1) [26, 27]. These structures are fundamental for cell adhesion to the substrate and the degradation of extracellular matrix, two processes involved in cancer progression. Both chronic and acute stimulation of KDELRs with artificial ligands enhances Src activation at focal adhesion and invadopodia, increases the number of invadopodia, and subsequently promotes the degradation of extracellular matrix (ECM) [26]. Along the molecular pathway connecting KDELR stimulation to ECM degradation, we identified the phosphorylation of Src substrates cortactin and ArfGAP with SH3 Domain, Ankyrin Repeat, and PH Domain 1 (ASAP1). Interestingly, both KDELR1 and KDELR2 were able to trigger ECM degradation, while KDELR3 was not [26].

**Fig. 2:**
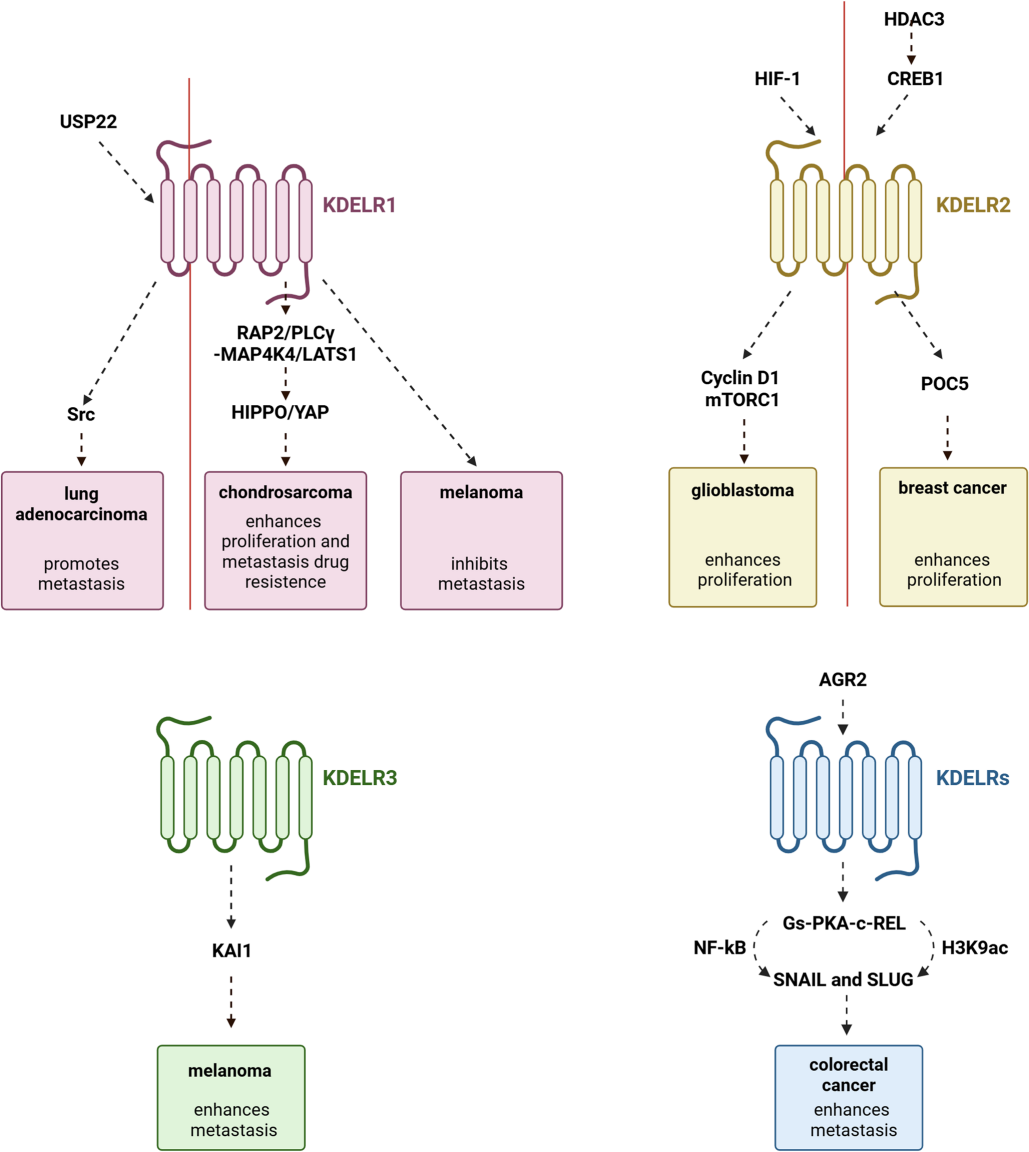
Involvement of KDELR family members across diverse tumor types. KDELR1 contributes to the progression of chondrosarcoma, lung adenocarcinoma, and melanoma by modulating the Hippo/YAP and Src signaling pathways. KDELR2 promotes the proliferation of glioblastoma and breast cancer cells by modulating the Cyclin D1/mTORC1 pathway and interacting with POC5. KDELR3 facilitates melanoma progression by downregulating the metastasis suppressor KAI1. KDELRs promote the progression of colorectal cancer by activating the Gs–PKA–c-Rel signaling pathway. The vertical line separates the subsets of upstream regulators and downstream effectors associated with the same receptor isoform

KDELR1 also controls cellular adhesion process by modulating the ECM composition [28]. Notably, KDELR1-deficient cells exhibit reduced adhesion under standard conditions, while their adhesion is significantly enhanced on collagen- or laminin-coated substrates [28]. This effect may be attributed to a compensatory upregulation of adhesion-related proteins (e.g. adhesion molecule 1, contactin-3, platelet glycoprotein 4, homeobox protein Mohawk, protocadherin-17, protocadherin β−4, protocadherin β−5, prostaglandin G/H synthase 2, tyrosine-protein kinase SYK, and T-lymphoma invasion and metastasis-inducing protein 1) in an attempt of the KDELR1-knockout cells to compensate for a dysfunctional extracellular matrix (ECM) [28]. Furthermore, KDELR2 has been reported to be overexpressed in metastatic non-small cell lung cancer (NSCLC) cells and bladder cancer, where it enhances Golgi-mediated release of matrix metalloproteinases (MMPs) to drive ECM invasion (Fig. 2) [29, 30]. Consistent with this, KDELR2 knockdown reduced metastasis without impacting cellular proliferation [29]. The involvement of G proteins was not investigated in these reports, although their potential role cannot be excluded.

In 2003, Velasco and co-workers demonstrated that the retrotransport of chaperones via KDELR requires PKA-dependent phosphorylation of serine 209 on the KDELR itself [31]. A few years later, our laboratory discovered that activating the KDELR through traffic synchronization or by using artificial KDEL ligands (HRP-KDEL and Bodipy-KDEL) stimulates PKA activity (Fig. 1) [32]. This signalling involves the classical Gαs subunit of heterotrimeric G protein, which activates adenylyl cyclase, leading to an increase in cAMP levels that control PKA (Fig. 1). It appears that the Golgi membranes host the whole PKA signalling pathways previously established on the plasma membrane (Table 1). By exploiting the autoactivation properties of overexpressed KDELRs, we provided evidence that KDELR1 is the primary activator of this signalling pathway, whereas KDELR2 exhibits lower activity, and KDELR3 is inactive [32]. This signalling pathway regulates the retrograde transport of KDELR from the Golgi to the ER and, more generally, the transport of Golgi membranes, involving the phosphorylation of over 200 proteins primarily associated with actin remodelling (Fig. 1). Among the key players downstream of this KDELR-PKA are LIM kinase and cofilin [32]. The role of PKA in the endomembrane circulation of KDELR was further confirmed by Yue et al., who demonstrated that ACBD3 is a key controller of the retrograde transport of KDELR by negatively regulating PKA [33]. Finally, KDELR activation via-PKA induces a repositioning of lysosomes as well as activation of autophagic flux and lipid droplet turnover (Fig. 1) [39]. This KDELR-dependent coordination of lipid metabolism is necessary to sustain secretion. Interestingly, only KDELR1 and partially KDELR2 can coordinate this action [39].

**Table 1:** Cell signalling activated by KDELRs

Receptor	Experimental Systems: Cellular, Human, and Animal Models	Signalling Pathways and Downstream Effects	References
KDELRs	- HeLa and HT-1080 cells	ACBD3-dependent regulation of KDELR-PKA interaction → Prevents cargo-free KDELRs from engaging in retrograde transport	[33]
KDELRs	- LoVo, SW480, HT-29, DLD-1, SW48, HCT116 and CT26 colorectal cancer cells	AGR2-KDELRs interaction activates Gαs-PKA-c-REL signalling pathway → Upregulates SNAIL and SLUG transcription factors	[34]
KDELRs	- *Drosophila* lines - N2a, HeLa, BE(2)C, S2 and Sf9 cells - mouse cortical neurons	KDELRs activation of Gαo-Rab1a/Rab3a signalling pathway → Regulates membrane trafficking and plasma membrane protrusions	[35]
KDELRs	- HeLa cells	KDELRs activation of Gα_q/11_-Src signalling pathway → Regulates anterograde membrane trafficking through the Golgi complex	[24]
KDELRs	- HF, HeLa and COS-7 cells	KDELRs activation of Src family signalling pathway → Regulates anterograde membrane trafficking through the Golgi complex	[22]
KDELRs	- HeLa cells	KDELRs activation of p38 MAPK kinases and JNKs → Affects cell fate upon ER stress	[13]
KDELR1	- HeLa and HT-1080 cells	KDELR1 activation of EGFR-STAT3 signalling pathway → Induces cell proliferation and migration	[36]
KDELR1	- Human samples: osteochondroma and chondrosarcoma - HEK293T, CHON-001, SW1353 and Hs 819.T cells	KDELR1 regulates Hippo-YAP1 signalling pathway via integrin-α3/5β5-PLCγ-MAP4K4 axis → Regulates malignant phenotypes and drug resistance in chondrosarcoma cells	[37]
KDELR1	- NCI-H1299, NCI‐H1975 and PC9 LUAD cells	KDELR1 activation of Src signalling pathway → Promotes proliferation, invasion and migration of LUAD cells	[38]
KDELR1	- HeLa and H4 cells	KDELR1 activation of Gαs-PKA pathway → Promotes lysosome repositioning to the Golgi area through DynLRB1 and p62/SQSTM1 phosphorylation	[39]
KDELR1	- N-ethyl-N-nitrosourea (ENU)-induced mutant mouse strain (T-red mice) - T cells from T-red mice	KDELR1 activation of the PP1–eIF2α signalling axis → Maintains T cell homeostasis through modulation of the integrated stress response	[40]
KDELR2	- Human samples: breast cancer tissues and corresponding paracancerous tissues - MDA-MB‐231, T47D, MCF‐7 and Hs 578 T breast cancer cells	KDELR2 increases POC5 expression → Promotes breast cancer cell proliferation	[41]
KDELR2	- U251, U-87, U373 and A-172 glioma cells	KDELR2 upregulates cyclin D1 expression → Promotes glioma cell proliferation	[42]
KDELR2	- Human samples: glioblastoma tissues and corresponding paracancerous tissues - LN229 and T98G glioblastoma cells	KDELR2 activation of mTORC1 signalling pathway → Promotes glioma cell proliferation	[43]
KDELR2	- A375MM and HeLa cells	KDELR2 activation of Src and FAK at focal adhesions and invadopodia → Promotes ECM degradation	[27]
KDELR3	- Mouse model of melanoma - Melanoblasts and melanocytes from the mouse model	KDELR3 interaction with gp78 regulates KAI1 protein levels and glycosylation → Promotes metastasis in melanoma	[44]
KDELR1 and 2	- A375MM and MDA-MB-231 cells	KDELRs activation of Src-cortactin and ASAP1 at invadopodia → Promotes ECM degradation	[26]
KDELR1 and 2	- COS-7, HeLa and CHO cells	KDELRs activation of Gαs-PKA pathway involving AC9, PDE7A1 → Regulates retrograde membrane trafficking from the Golgi to the ER → Upregulates membrane trafficking–related genes through CREB1 activation	[32]

The relevance of the KDELR-Gα subunit axis is further supported by data involving Gαo, in addition to Gαq and Gαs. Specifically, Solis et al. showed that KDELR and Gαo colocalize on the Golgi membranes and interact in pull-down experiments [35]. The stimulation of KDELR by different means (synthetic KDEL peptide and transfection with KDEL containing proteins) promoted the loading of GTP on Gαo (Fig. 1). It appears that the relationship between KDELR and Gαo closely resembles the activation of heterotrimeric G proteins by GPCRs. However, unlike the classical paradigm, the beta/gamma complex is not part of the signalling complex [35]. Finally, the KDELR activation of Gαo favour the dissociation of guanine nucleotide dissociation inhibitor (GDI) complexed from Rab1a/Rab3a that in turn can be activated by guanine nucleotide exchange factors (GEFs) [35]. This signalling is thought to coordinate the arrival of material from the secretory pathway with the growth of protrusion on the plasma membranes (Fig. 1) [35]. In fact, activation of KDELR through Gαo enhances both the length and number of neurites in N2A cells, as well as protrusions in Drosophila S2 cells. This activity was confirmed in vivo, as transgenic Drosophila overexpressing KDELR and Gαo synergistically stimulate the multiple-wing-hairs (mwh) phenotype [35]. Unfortunately, the specific KDELR isoform used in this study remains unclear.

## Crosstalk between KDELR and EGF receptor

As mentioned above, a portion of KDELRs was detected on the plasma membrane, cycling via clathrin-mediated endocytosis to the endosomes, then to the Golgi, and finally returning to the cell surface [6]. The extracellular ligands that activate the receptors on the plasma membrane are likely proteins with the C-terminal KDEL motif, such as mesencephalic astrocyte-derived neurotrophic factor (MANF), ERp57 and BiP [36, 45]. Several studies reported the presence of KDEL-containing chaperones on the plasma membrane, particularly in cancer cells [46]. Regarding the potential signalling triggered by chaperones and KDELRs at the plasma membrane, a recent study demonstrated that KDELR1 interacts with the EGF receptor and is internalized in complex with it [36]. Actually, it is the EGF receptor, which contains the di-leucine motif absent in KDELRs, that interacts with AP2 and drives the internalization of the complex KDELR1-EGFR via clathrin-coated vesicles. It was demonstrated that the binding of KDEL ligands to KDELR1 induces its oligomerization, enhances its interaction with EGFR, and promotes their internalization [36]. The crosstalk between KDELR1 and EGFR also leads to EGFR activation and STAT3 phosphorylation. This KDELR1-initiated signalling stimulates cell proliferation and motility, explaining how KDEL chaperones released by cancer cells can influence cancer progression. Notably, this signalling cascade does not involve heterotrimeric G proteins [36].

## Exploring KDELRs in cancer through colorectal and mammary tumour examples

The reported signalling pathways and cellular functions suggest that KDELRs play a role in cancer biology, including cell proliferation and invasion. In tumours, rapid cell proliferation, increased cellular metabolism, and insufficient vascularization lead to acidosis, along with decreased glucose and oxygen levels. This, combined with elevated protein synthesis, genomic instability, and oxidative stress, leads to the accumulation of misfolded proteins [47, 48]. ER stress and activation of the UPR have been reported in many cancers and are implicated in the migration, homing, and invasion of cancer cells [47, 49, 50].

Among the UPR- and cancer-upregulated chaperones is anterior gradient-2 (AGR2), a member of the protein disulfide isomerase (PDI) family, featuring a KTEL C-terminal sequence [51]. AGR2 acts as an oncogene and controls the EMT process in multiple tumours [34]. During the EMT process, epithelial cells acquire migratory and invasive abilities by loss of epithelial markers and gain of mesenchymal markers [52]. EMT is regulated by multiple EMT-activating transcription factors (EMT-TFs), including SNAIL, SLUG, TWIST, and ZEB [52]. There is evidence that AGR2 can regulate EMT in colorectal cancer through its interaction with KDELRs, owing to its KTEL retention motif (Fig. 2) [34]. In colorectal cancer cells, AGR2 overexpression can be stimulated by prostaglandin E2 (PGE2), released by tumour-infiltrating macrophages, which activates the EP4–PI3K–AKT signalling pathway. AGR2 interacts with all three KDELRs, and their activation triggers the Gs-PKA pathway, which subsequently induces the upregulation of the transcription factors SNAIL and SLUG by increasing c-Rel within the NF-κB signalling pathway [34]. Additionally, the AGR2-KDELR-Gs-PKA pathway enhances the acetylation of histone 3 at lysine 9 (H3K9ac) within the promoters of SNAIL and SLUG, further promoting their upregulation [34].

Epigenetic alterations are frequently involved in tumorigenesis and cancer progression [53]. One key mechanism of epigenetic regulation involves the activation of histone deacetylases (HDACs), and aberrant expression of HDACs has been reported in various cancers [54]. Inhibition of HDACs using HDAC inhibitors (HDACi) represents a promising therapeutic strategy for cancer treatment [55]. In breast cancer, HDAC3 is upregulated in tumour tissues, and KDELR2 has been identified as a target of this deacetylase [41]. Specifically, HDAC3 elevates the levels of cAMP-response element binding protein (CREB1), a transcription factor that regulates the expression of KDELR2. In turn, increased KDELR2 promotes breast cancer progression by binding and stabilizing the centrosomal protein 5 (Fig. 2) [41].

## KDELR2 carry out a key role in glioblastoma

Glioma is the most common malignant tumour in the central nervous system. Glioblastoma multiforme (GBM) is high-grade of glioma and is the most aggressive brain tumour with an overall survival of 15 months [56]. To date, treatment protocols include maximal safe surgical resection, radiation therapies and chemotherapy. In GBM the therapeutic effect of treatments is greatly limited by the blood-brain barrier which makes it difficult for many chemotherapy agents to cross [57]. Temozolomide (TMZ), the most commonly used chemotherapeutic agent, extends median patient survival by only 14.6 months [57].

GBM, as other solid tumours, exhibits ER stress and UPR [21, 58]. Multiple studies have shown that KDELR2 is highly expressed in GBM tissues and that its expression levels are negatively correlated with glioma patient prognosis (Fig. 2) [52, 59], suggesting its potential as a prognostic marker [42, 43, 59]. Furthermore, in vitro studies with GBM cell lines showed that KDELR2 down-regulation inhibits cell proliferation and tumour growth [42, 43] while significantly increasing apoptosis [59]. KDELR2 knockdown enhances glioma cell apoptosis through multiple mechanisms, including activation of the CHOP and JNK/p38 pathways [59]. Additionally, in subcutaneous xenograft mouse models, KDELR2 knockdown in GBM cells leads to slower tumour proliferation, resulting in significantly reduced tumour weight and volume (Fig. 2) [42, 43]. These effects were attributed to KDELR2’s regulation of the cell cycle, which occurs through the modulation of Cyclin D1 expression and the activation of the mTORC1 pathway [42]. GBM tissues are characterized by extensive necrotic and hypoxic regions, along with constitutive expression of hypoxia-inducible factor 1 (HIF-1), the key regulator of the cellular response to hypoxia [60]. Interestingly, it has been reported a positive correlation between the expression levels of HIF-1 and KDELR2 [43]. In fact, the promoter region of KDELR2 contains one hypoxia response elements (HRE) thus resulting directly regulated by HIF-1 [43]. Moreover, KDELR2 expression was positively correlated with Th2 cells infiltration in GBM [61]. Finally, gene expression studies have shown that KDELR1 is also overexpressed in gliomas, with its expression positively correlating with tumour grade, recurrence, necrosis, isocitrate dehydrogenase (IDH) mutations, reduced overall survival, and increased infiltration of T cells and macrophages [62].

## KDELR1 and KDELR3 play opposing roles in melanoma

The role of KDELRs have also been investigated in melanoma. Melanoma is an aggressive tumour of the skin, induced from malignant transformation of melanocytes and with a high tendency to metastasize. The number of melanoma cases among the elderly (> 65 years) has been rising steadily, accounting for more than 300,000 new cases and almost 6000 deaths occurring in 2020 [63]. In most cases, melanoma patients have local disease that can be treated successfully with surgery alone [64]. KDELR1 and KDELR3 play an oppositive role during melanoma progression [44]. In particular, KDELR3 is upregulated in malignant melanoma, is localized at both the cis- and trans-Golgi compartments of metastatic melanoma cells and plays a key role in the metastatic process (Fig. 2) [44]. KDELR3-knockdown melanocytes have an enhanced sensitivity to ER stress, suggesting an important role of this receptor as a promoter of metastatic cell survival in both mouse and human melanoma cells [44]. Furthermore, the overexpression of the receptor enhances the anchorage-independent growth of melanoblasts [44]. KDELR3 knocked-down melanoma cell lines in the experimental tail vein metastasis assay showed reduced metastatic potential, with no appreciable difference in subcutaneous in-vivo tumour growth (Fig. 2) [44]. At the molecular level, KDELR3 plays a regulatory role in regulating the expression and post-translational modification of the tetraspanin glycoprotein CD82/KAI1 [44]. KAI1 is a well-documented metastasis suppressor in melanoma and other tumours by mediating interactions between extracellular and intracellular molecules at the cell membrane [65, 66]. KDELR3 knockdown leads to the accumulation of the highly glycosylated form of KAI1, which is associated with inhibition of cell motility and a reduction in metastatic progression [44]. Interestingly, KDELR1 plays an opposing role in melanoma progression, with its knockdown promoting metastasis, suggesting that this receptor functions as an inhibitor of melanoma metastasis (Fig. 2) [44]. Finally, KDELRs have been identified as potential biomarkers in uveal melanoma [67–69]. KDELR2 and 3 expression levels correlated with immune infiltration of macrophages and T cells in uveal melanoma (Fig. 2; Table 2). High levels of KDELR3 expression are associated with reduced disease-free survival and poor overall prognosis in this malignancy [67].

**Table 2:** KDELR upregulation correlates with immune cell infiltration in tumours

Receptor	Cancer type	Effect on immune cells	References
KDELRs	- Lung Adenocarcinoma	→ Associated with downregulation of CTLA-4 → Reduces CD8+ T cell infiltration	[70]
KDELR1	- Head and neck squamous cell carcinoma	→ Correlated with cancer associated fibroblasts → Reduces CD8+ T and B cell infiltration	[71]
KDELR1	- Glioma	→ Associated with CD4+ T and CD8+ T cell infiltration	[62]
KDELR2	- Bladder cancer	→ Associated with macrophage infiltration (M2 polarization and immunosuppression)	[72]
KDELR2	- Bladder urothelial carcinoma	→ Associated with macrophages, neutrophils and Th2 cell infiltration	[73]
KDELR2	- Uveal melanoma	→ Associated with CD8+ T cell infiltration by antigen processing and cytokine-cytokine receptor interaction	[69]
KDELR2	- Glioblastoma	→ Associated with infiltration of macrophages, dendritic cells and Th2 cells	[61]
KDELR3	- Glioma	→ Associated with infiltration of macrophages, Th2 cells, neutrophils, eosinophils, immune dendritic cells, T cells, activated dendritic cells, Natural killer cells	[74]
KDELR3	- Uveal melanoma	→ Associated with M1 macrophage, CD8+ T, follicular helper and dendritic cell resting → Activation of CD4+ T cell memory	[67]
KDELR3	- Hepatocellular carcinoma	→ Associated with CD4+ T cell infiltration	[75]
KDELR3	- Bladder cancer	→ Associated with infiltration of various immune cells in hypoxia microenvironment	[76]

## KDELR1 is deeply involved in chondrosarcoma malignancy and lung adenocarcinoma

Chondrosarcomas are rare bone cancers that originate from cartilage-producing cells [77]. KDELR1 expression positively correlates with chondrosarcomas malignancy grade and lower overall survival of patients (Fig. 2) [37]. In vitro studies showed that downregulation of KDELR1 reduces cell migration and survival following cisplatin treatment [37]. With respect to the molecular pathway regulating these functions, KDELR1 plays a regulatory role in the HIPPO/YAP signalling pathway. Specifically, KDELR1 knockdown increases YAP1 phosphorylation, leading to its retention in the cytoplasm. This is accompanied by the downregulation of other HIPPO pathway components, including AMOT, CCN1, and ANKRD2 [37]. The observed upregulation of MAP4K4 and enhanced phosphorylation of LATS1 contribute to YAP1 inactivation in KDELR1-deficient cells. Additionally, the accumulation of integrin-α3/β5 in the ER and Golgi, along with elevated expression and phosphorylation of RAP2 and PLCγ, appears to drive MAP4K4 activation [37]. In summary, KDELR1 modulates chondrosarcoma cell behaviour by regulating the secretion of integrin-α3/β5, thereby influencing YAP1 activity via the RAP2/PLCγ–MAP4K4/LATS1 signalling axis.

A recent study also reported a role for KDELR1 in lung adenocarcinoma (LUAD) progression, highlighting its influence on both tumour cell behaviour and the tumour microenvironment (Fig. 2) [38]. siRNA knockdown and overexpression experiments revealed that KDELR1 promotes LUAD cell proliferation, invasion, and migration. In vivo, elevated KDELR1 expression was associated with poorer patient outcomes (Fig. 2) [38]. Although the molecular mechanisms are still not fully understood, Zhen et al. [38] showed that high expression of ubiquitin-specific protease 22 (USP22) in LUAD cells promotes Src activation downstream of KDELR1. From a molecular standpoint, USP22 regulates KDELR1 expression by preventing its degradation through the proteasome. It enhances receptor stability by removing K48-linked polyubiquitin chains, which would otherwise target KDELR1 for degradation. The resulting activation of Src, in turn, promotes the formation of invadopodia and ultimately drives metastasis. Consistent with this, KDELR1 knockdown was shown to reduce Src activation and reverse the metastatic effects of USP22, thereby confirming a direct link between these molecules and poor prognosis in LUAD patients [38].

## KDELR1 modulates the immune response

**Fig. 3:**
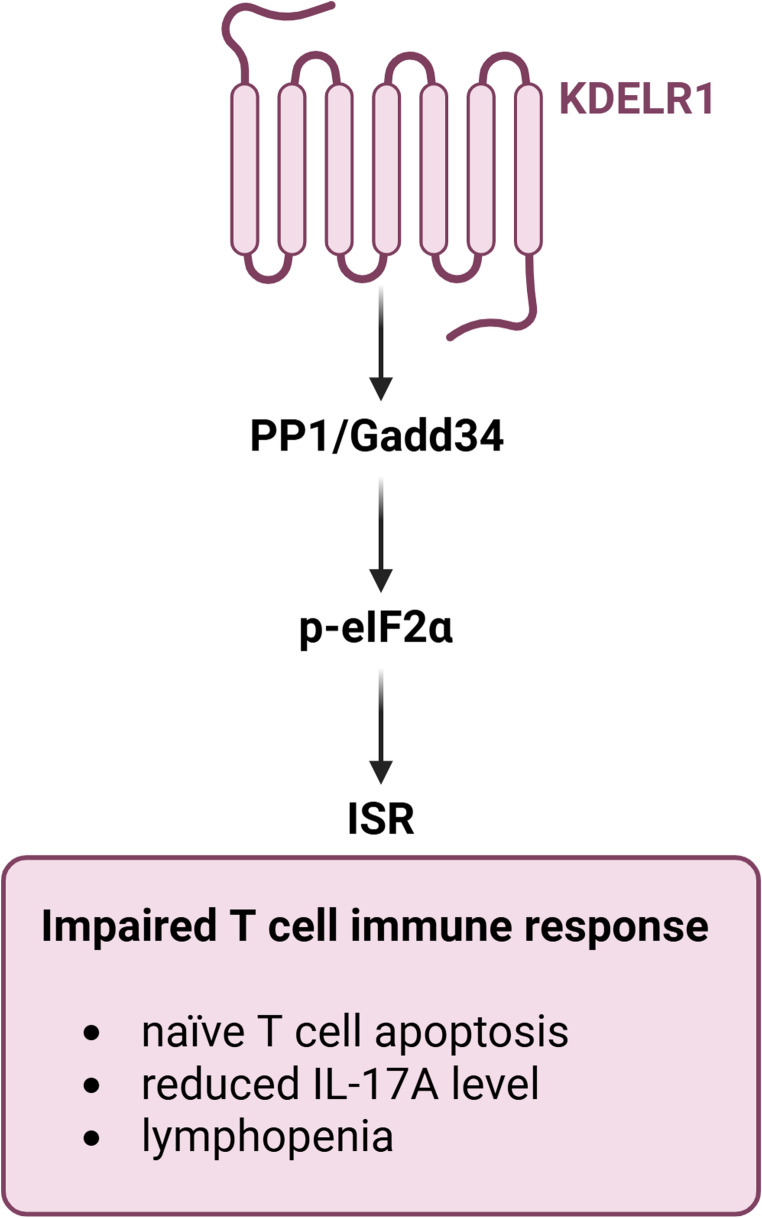
KDELR1-mediated regulation of immune responses. In vitro and in vivo data have shown that disrupted KDELR1 signalling impairs immune T cell regulation, leading to increased apoptosis of naïve T cells, reduced IL-17 A production, and lymphopenia. These effects highlight the role of KDELR1 in maintaining T cell homeostasis

The importance of KDELRs in tumour biology is further underscored by the role of KDELR1 in shaping the immune system (Fig. 3) [78]. In 2015, Kamimura et al. to investigate naïve T-cell homeostasis generated a T-red mice clone by N-ethyl-N-nitrosourea (ENU) mutagenesis [40]. These mice exhibited a reduced number of naïve T cells and carried a Ser123Pro variant of KDELR1. Complementation experiments with the wild-type KDELR1 successfully rescued the phenotype, while KDELR1 knockout mice mimicked the T-red phenotype, demonstrating that this receptor is directly responsible for the observed phenotype [40]. These animals also displayed a reduced T cell response, as evidenced by lower IL-17 A levels in a collagen-induced arthritis model, and showed decreased antibody levels following immunization with ovalbumin (Fig. 3) [40]. As it pertains to the molecular mechanism, naïve T cells underwent apoptosis following activation of integrated stress response (ISR) and BIM overexpression. Among the genes affected by activated ISR in these animals were ASNS, CHOP, TRIB3, and VEGFA [40]. ISR was triggered by the reduced interaction between KDELR1 and the protein phosphatase PP1/Gadd34, which plays a key role in the dephosphorylation of the translation factor eIF2α. The weak interaction between the receptor and PP1/Gadd34 reduces phosphatase activity, impairing the maintenance of protein synthesis homeostasis in the ER. This triggers ISR, ultimately leading to T cell apoptosis. The naïve T cells that survive in the absence of functional KDELR1 are likely those partially stimulated by the T cell receptor, potentially engaged by self-antigens [40]. An independent study investigating the *Daniel Gray* mouse line, generated by ENU mutagenesis, revealed lymphopenia and a reduced ability to clear viral infections [79]. More specifically, *Daniel Gray* mutant mice exhibit hypopigmentation, reduced thymic cellularity, and decreased percentages of CD4⁺ and CD8⁺ T cells in the blood. These features make them a valuable model for studying T cell development and protein trafficking [79]. Molecularly, T cells from *Daniel Gray* mice exhibited overexpression of CD44 in both CD4+ and CD8+ T cells, along with reduced T cell receptor expression (Fig. 3) [79]. Genotypic characterization identified a Tyr158Cys missense mutation within KDELR1, and KO-KDELR1 mice exhibited a similar phenotype. The specific mechanisms disrupted by the loss of KDELR1 in this context remain largely unclear, apart from the involvement of FAS in T cell death [79].

## Conclusions

**Fig. 4:**
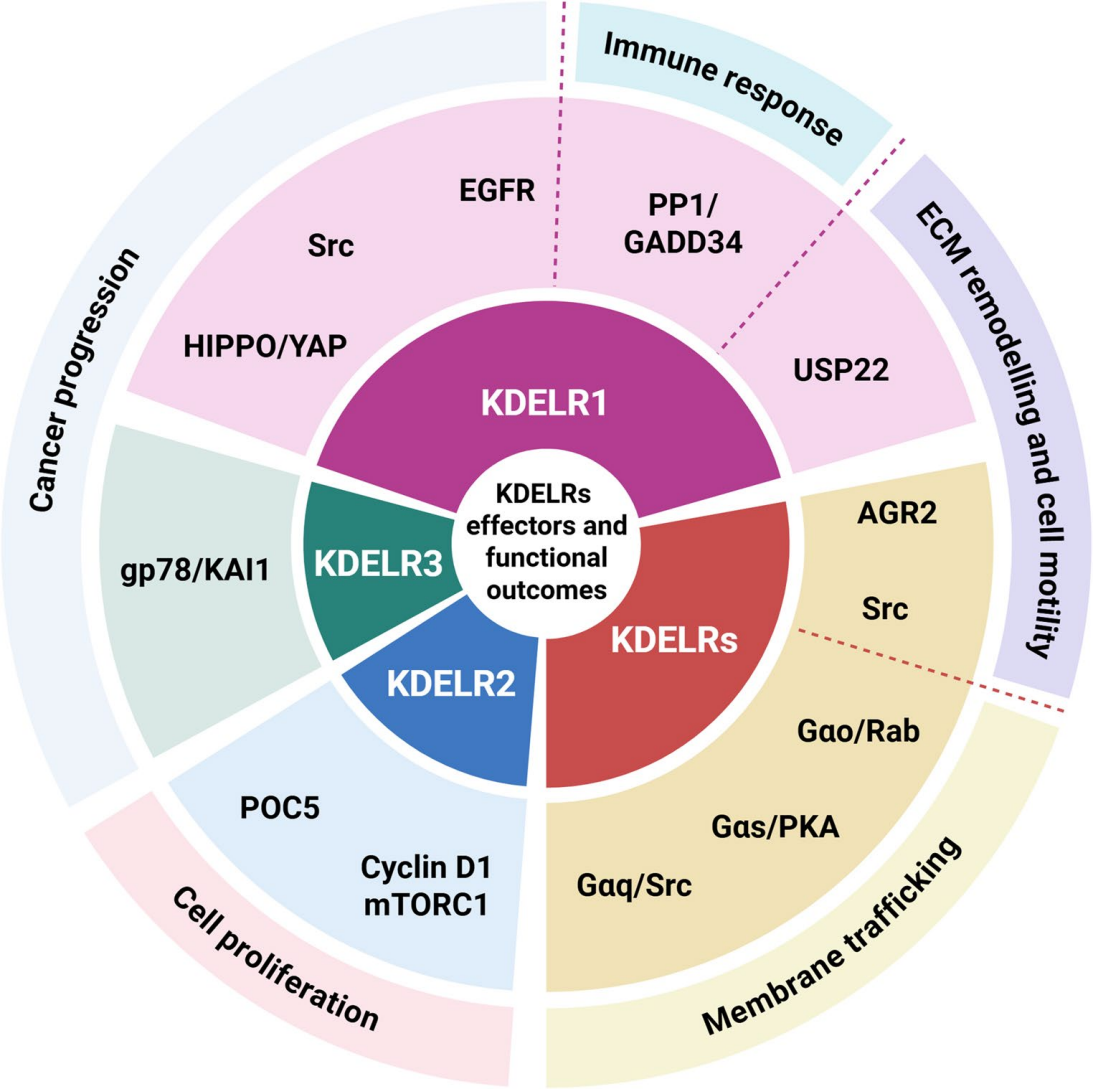
Circular schematic summarising KDELR downstream effectors and their main associated functions. The diagram illustrates the roles of KDEL receptors in various cellular processes, highlighting the distinct functions of KDELR1, KDELR2, and KDELR3, along with their respective downstream effectors. The innermost layer depicts the KDELRs themselves; the middle layer shows their associated effectors and signaling pathways; and the outermost layer illustrates the resulting cellular processes and broader functional outcomes shaped by these interactions

KDELR was discovered over 40 years ago in yeast as a protein primarily responsible for retrieving ER chaperones [7]. However, it was hypothesized that it might have additional roles, given that yeast cells could not grow in the absence of KDELRs [80]. During evolution, the KDELR gene underwent duplication, giving rise to a small family of three members in vertebrates [3]. The KDELR family share a high degree of amino acid identity (65–83%), and even greater homology (up to 94% between KDELR1 and KDELR2), yet its members have acquired distinct functional roles (Figs. 2, 3 and 4; Tables 1 and 2). This raises the question of how KDELR isoforms can perform distinct and non-redundant functions. It is likely that their functional specificity is determined by a small set of isoform-specific amino acid residues. For example, the most similar isoforms, KDELR1 and KDELR2, differ by 35 amino acids, variations that may affect both affinity for luminal ligands and interactions with cytosolic partners.

Over time, KDELRs have evolved important signalling functions by integrating into classical signalling pathways and interacting with key signalling molecules such as p38MAPK, PKA, Src and EGFR [13, 22, 32, 36]. KDELR signalling influences fundamental cellular processes such as membrane trafficking and the UPR, as well as more complex events including cell proliferation and ECM remodelling (Fig. 4) [1, 26, 27, 36, 41, 80, 81].

Such activities can potentially affect various physiological functions, although their role in cancer has been more extensively studied. We would like to underscore that the regulatory functions of KDELRs in membrane trafficking and signalling likely act in concert to promote cancer progression. Distinguishing the specific contributions of each function remains a significant challenge. Furthermore, based on available data, the role of KDELRs in cancer progression appears to be correlative rather than causative, as no current studies have demonstrated that they function as a primary driving force. Nevertheless, KDELRs clearly play a significant role in the progression of the disease.

KDELR signalling is closely associated with cancer progression, not only through its direct effects on cancer cells (Table 1) but also by modifying the tumour microenvironment via ECM degradation and immune system regulation (Table 2). Specifically, the expression of all three KDELRs in cancer cells has been associated with immune cell infiltration across various tumour types (Table 2), with KDELR1 also recognized as a modulator within immune cells themselves [40, 79]. Based on the above, we strongly believe that achieving a comprehensive understanding of KDELR signalling could pave the way for innovative approaches to cancer treatment.

## Data Availability

No data was generated as part of this paper.
